# Irreversibility of Pressure Induced Boron Speciation Change in Glass

**DOI:** 10.1038/srep03770

**Published:** 2014-01-20

**Authors:** Morten M. Smedskjaer, Randall E. Youngman, Simon Striepe, Marcel Potuzak, Ute Bauer, Joachim Deubener, Harald Behrens, John C. Mauro, Yuanzheng Yue

**Affiliations:** 1 Section of Chemistry, Aalborg University, DK-9000 Aalborg, Denmark; 2 Science and Technology Division, Corning Incorporated, Corning, NY 14831, USA; 3 Institute of Non-Metallic Materials, Clausthal University of Technology, 38678 Clausthal-Zellerfeld, Germany; 4 Institute of Mineralogy, Leibniz University Hannover, 30167 Hannover, Germany

## Abstract

It is known that the coordination number (CN) of atoms or ions in many materials increases through application of sufficiently high pressure. This also applies to glassy materials. In boron-containing glasses, trigonal BO_3_ units can be transformed into tetrahedral BO_4_ under pressure. However, one of the key questions is whether the pressure-quenched CN change in glass is reversible upon annealing below the ambient glass transition temperature (*T*_g_). Here we address this issue by performing ^11^B NMR measurements on a soda lime borate glass that has been pressure-quenched at ~0.6 GPa near *T*_g_. The results show a remarkable phenomenon, i.e., upon annealing at 0.9*T*_g_ the pressure-induced change in CN remains unchanged, while the pressurised values of macroscopic properties such as density, refractive index, and hardness are relaxing. This suggests that the pressure-induced changes in macroscopic properties of soda lime borate glasses compressed up to ~0.6 GPa are not attributed to changes in the short-range order in the glass, but rather to changes in overall atomic packing density and medium-range structures.

Any natural or man-made material will exhibit a significant change in microstructure and properties when subjected to high pressure^1^,^2^,^3^,^4^,^5^; particularly the coordination number (CN) of atoms or ions will generally increase with pressure^6^,^7^,^8^,^9^. It has long been known that when a glassy material or its liquid state is subjected to sufficiently high pressure, significant changes can take place in the local and medium-range structure, vibrational density of states, and physical properties^4^,^5^,^6^,^7^,^8^,^10^,^11^. In recent years, theoretical modeling approaches have provided insights regarding the structural response of various non-crystalline materials to composition, temperature, and pressure^11^,^12^,^13^,^14^,^15^. Nevertheless, the relationship between structure and dynamics in glass and glass-forming melts under high pressures remains a challenging problem in condensed matter science. The difficulties in addressing this problem are due to both experimental limitations at high pressures and the inadequacy of computer simulations under such conditions. It is crucial to determine the link between the microscopic structure and macroscopic properties of glasses under high pressure from both scientific and technological perspectives, since the glass structures frozen-in under elevated pressure may give rise to properties unattainable under ambient pressure. Borate glass is the quintessential example of glass capable of dramatic changes in short-range order as a function of composition, pressure, and thermal history, and hence we select a simple ternary borate system, namely, sodium-calcium-borate glass, for this study in order to address the above mentioned challenging problem. Boric oxide (B_2_O_3_) is also widely used as a network forming constituent in many high-tech glass materials owing to its contribution to high glass forming ability and low melting temperature, and for its favorable impact on thermal, mechanical, and optical properties.

One of the most striking structural features of borate glasses is the transformation of the coordination number (CN) of boron from three to four upon compression^16^,^17^,^18^,^19^, which is associated with anomalous pressure dependence of viscosity^6^,^16^ and topological disorder^8^,^12^. Densification of borate glasses can be achieved by applying isostatic pressure to glass directly at room temperature^16^,^17^, or by pressure-quenching from the molten state^18^,^19^,^20^,^21^. However, the elastic part of the densification relaxes during pressure release, i.e., the density of a compressed glass at ambient pressure decreases to the value before compression upon reheating below the glass transition temperature (*T*_g_)^17^. For vitreous B_2_O_3_, it has been shown that the fraction of boroxol rings decreases with increasing pressure^16^,^20^,^22^, leading to densification of the glass^16^ and increase of elastic moduli^20^. When the pressure applied at room temperature is released, such densification is irreversible since the local structures may be topologically/stereochemically unfavorable for the reformation of boroxol rings^16^. However, it is not clear whether this irreversibility of densification is linked to the CN change of boron^23^. More recently, it has been found that the BO_3_ fraction suddenly drops at pressures just above 4 GPa and then approaches zero as the pressure is further increased^17^. A structural response of vitreous B_2_O_3_ to pressure below 4 GPa was not detected. After decompression from high pressure the boron coordination reverts back from tetrahedral to trigonal, however, the densification is apparently permanent^17^. It has been attempted to correlate these changes in microscopic structure with the macroscopic properties. For example, the viscosity of the B_2_O_3_ liquid along the melting curve has been shown to decrease by 4 orders of magnitude upon a pressure increase up to 5.5 GPa but then remains unchanged upon further increase of the pressure^22^. However, a generally accepted viewpoint about the microscopic origin of the pressure-induced changes in the macroscopic properties is still lacking.

The abovementioned studies were carried out on vitreous B_2_O_3_ at room temperature and rather high pressure up to 22.5 GPa. It should be noted that the structure of glasses compressed at room temperature (in diamond anvil cell) is different from that obtained by pressure-quenching liquids from above *T*_g_^21^, even though the main structural changes upon compression (i.e., decreased fraction of boroxol rings and increased fraction of tetrahedral boron) are identical. It should also be noted that modified borate and pure B_2_O_3_ glasses have different initial concentration of boroxol rings and may exhibit different densification mechanisms. However, Lee *et al.* have shown that the pressure dependent structural changes of a lithium borate glass are similar to those of vitreous B_2_O_3_^24^. In particular, they showed that the pressure-induced CN change in Li_2_B_4_O_7_ glass from three-coordinated to four-coordinated boron at room temperature begins around 5 GPa and the BO_4_ fraction increases with pressure from about 50% at 1 atm to more than 95% at 30 GPa.

In this work we investigate the microscopic and macroscopic responses of the soda-lime borate glass to comparatively *low* pressure at an *elevated* temperature around the *T*_g_, since numerous high pressure studies at room temperature have already been reported in literature and it is also our aim to detect the sensitivity of glass structure and properties to the low pressure. Moreover, these conditions are chosen to be able to prepare compressed samples, which are large enough to allow for subsequent characterization of macroscopic properties. The as-produced glass has a nonzero equilibrium concentration of BO_4_ due to the presence of network modifiers (Na_2_O and CaO) that act to charge balance the tetrahedral boron species. Only recently has it been found that boron speciation (i.e. the CN) changes occur at rather low pressure (<0.6 GPa) near *T*_g_ and can be frozen-in under pressure^18^. Here the key questions arise: Is the pressure-quenched CN change reversible upon annealing at temperatures slightly below *T*_g_ at atmospheric pressure? Are the pressure-induced changes in the macroscopic properties also reversible upon annealing? What is the microscopic origin of the pressure-induced changes in the macroscopic properties? We answer these questions by investigating the structure and property responses of the sodium-calcium-borate glass to pressurization at <0.6 GPa and subsequent annealing at 0.9*T*_g_ under ambient pressure. We thereby hope to obtain a better understanding of the pressure-structure-property relationship of this and other glass systems, which can be used for tailoring both microscopic and macroscopic properties of glassy materials.

## Results

### Physical properties and heat capacity

**Figure 1:**
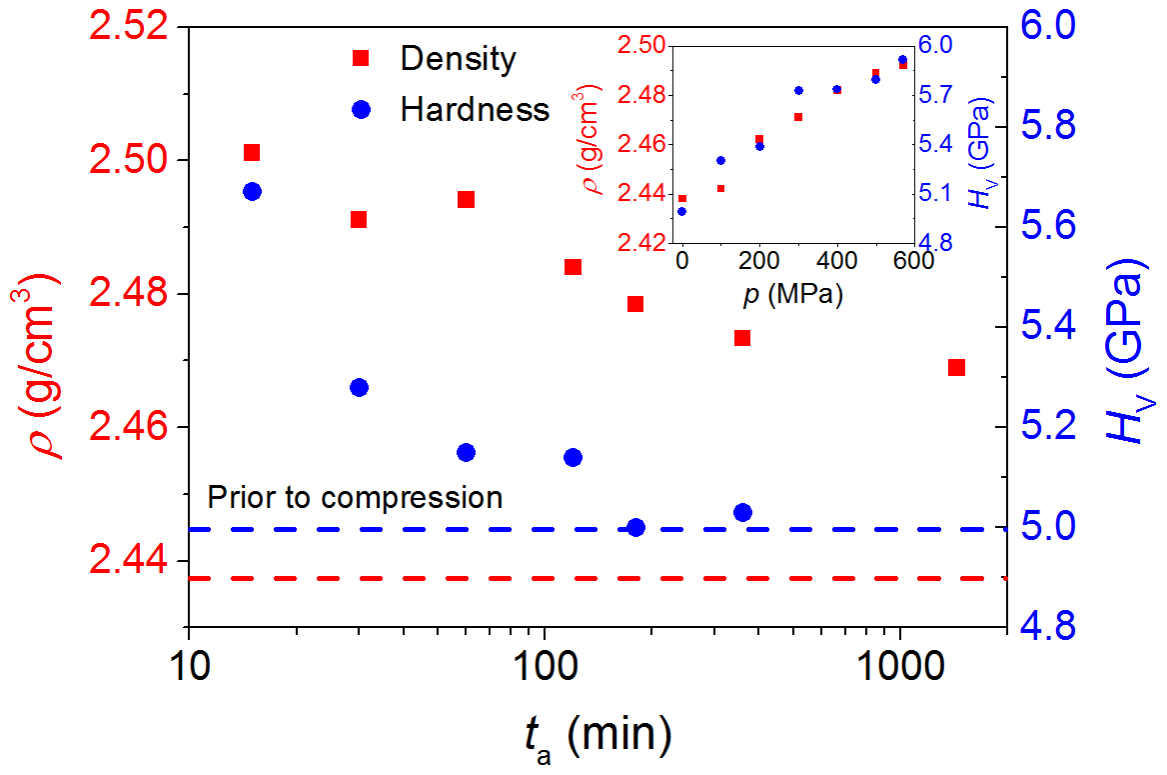
Relaxation of macroscopic properties. Evolution of density (*ρ*) and Vickers hardness (*H*_V_) with annealing duration (*t*_a_) at 0.9*T*_g_ = 688 K of the borate glass compressed at 570 MPa. The dashed lines indicate the values of *ρ* and *H*_V_ prior to compression. Inset: impact of isostatic pressure (*p*) on *ρ* and *H*_V_. The errors of *ρ* and *H*_V_ are around ±0.005 g/cm^3^ and ±0.2 GPa, respectively.

The glass composition under study is 25Na_2_O – 10CaO – 65B_2_O_3_ (mol%). As reported previously^25^, the density and Vickers hardness of this glass increase approximately linearly with increasing isostatic pressure (inset of Fig. 1). The glass isostatically compressed at 570 MPa is then annealed under ambient pressure at 688 K, i.e., 0.9 times its ambient *T*_g_ for various durations (*t*_a_). This results in a decrease of both density and Vickers hardness with increasing annealing duration (Fig. 1). In other words, these macroscopic properties of the compressed glass are relaxing during annealing towards those of the glass prior to compression. While Vickers hardness has essentially decreased to its original value prior to compression (5.0 GPa) after annealing for 6 h, density has only decreased to ~2.47 g/cm^3^ after annealing for 24 h, which is significantly higher than the value prior to compression (2.438 g/cm^3^), i.e., density has relaxed by only 43% of the total possible relaxation.

**Figure 2:**
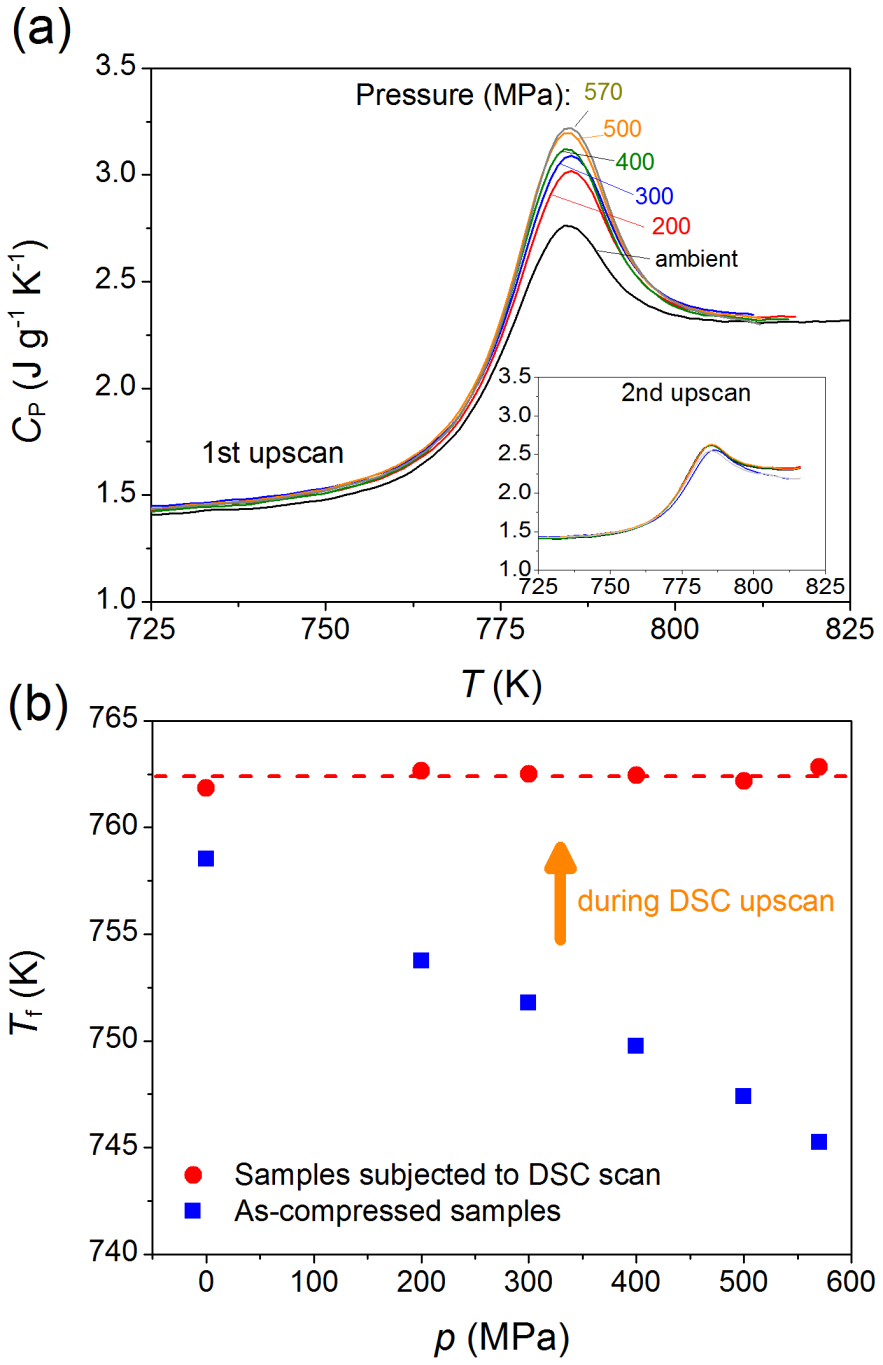
Pressure history dependence of calorimetric glass transition. (a) Evolution of endothermic overshoot in isobaric heat capacity (*C*_p_) at the glass transition for samples with different pressure history. DSC scans were performed at 10 K/min at ambient pressure. Inset: second DSC upscans at 10 K/min of all samples following prior up- and downscans at 10 K/min. (b) Pressure history dependence of the fictive temperature (*T*_f_) evaluated from the first and second DSC upscan, respectively.

With increasing isostatic pressure, the overshoot in the isobaric heat capacity (*C*_p_) above *T*_g_ increases as illustrated in Fig. 2a, where the evolution of *C*_p_ with temperature during the first DSC upscan is shown. This overshoot is considered to be a direct consequence of the nonexponentiality of the relaxation process^26^, i.e., due to broadening of the relaxation time distribution. This also implies that subjecting the glass to high pressure forces the glass into a lower region of the enthalpy landscape compared to the glass under ambient pressure, even though it should be noted that the enthalpy landscape itself is changing as a function of pressure. In agreement with previous studies^19^,^27^, we find that an increase in isostatic pressure enhances the nonexponentiality of the enthalpy relaxation as measured *ex situ*. With increasing pressure on the glass, the density of the glass increases, and at the same time the topological degree of atomic freedom decreases as well due to the BO_3_→BO_4_ conversion, i.e., the increase of network connectivity. Upon heating through the glass transition region, the glass compressed at higher pressures should exhibit a larger jump in configurational entropy in order to approach the liquid state. This is also shown in Fig. 2b, in which the pressure dependence of the fictive temperature (*T*_f_) is plotted. The procedure for determining *T*_f_ based on DSC data is described elsewhere^28^,^29^.

**Figure 3:**
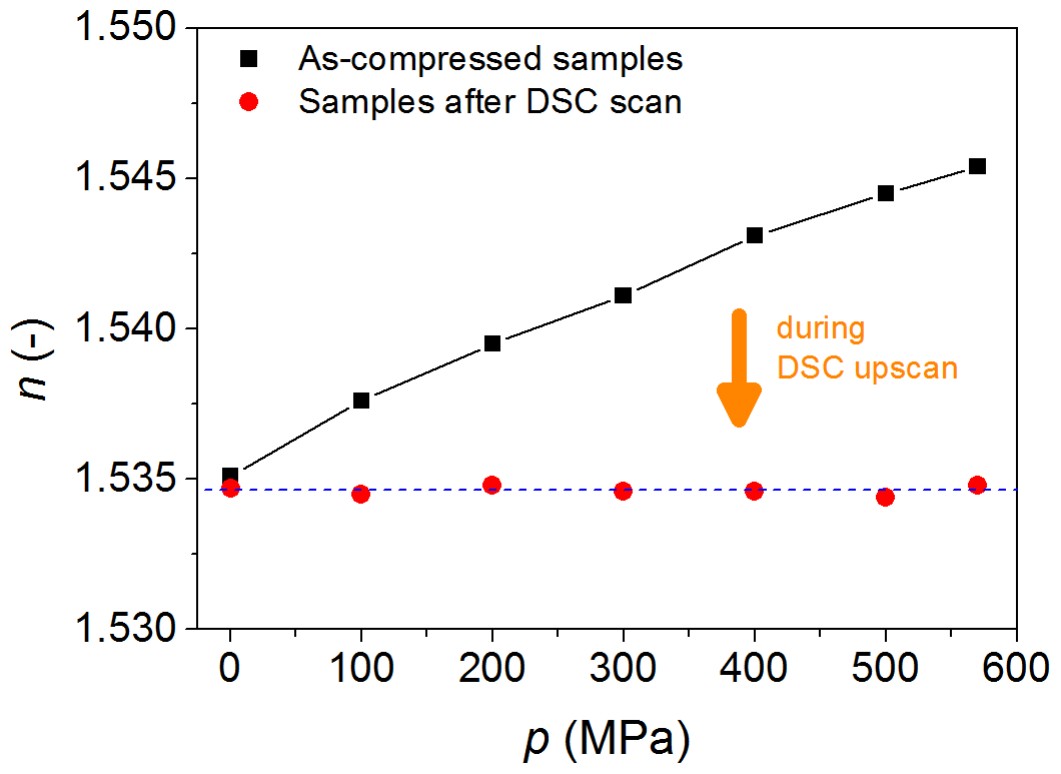
Recovery of refractive index during DSC scan. Pressure history dependence of the refractive index (*n*) at 633 nm before and after the first DSC scan, as shown in Fig. 2a.

The compressed glass relaxes in the glass transition region during the first upscan and recovers back to its original state with respect to enthalpy, i.e., that of the uncompressed glass. In other words, the enthalpy level of the glass reaches that of the glass cooled under standard conditions, e.g., at 10 K/min and ambient pressure. This is seen from the second DSC upscans as shown in the inset of Fig. 2a and in Supplementary Figure S1. Enthalpy recovery of the compressed glass during the DSC upscan is a “structural depression” process, induced by thermal excitation under ambient pressure^27^. The structural depression leads to an increase in enthalpy, which is manifested as an enhancement of the overshoot during the first DSC upscan. This is also evident from the relaxation of refractive index before and after the first DSC upscan (Fig. 3). With increasing isostatic pressure, the refractive index at 633 nm increases due to densification, but the measured value on the sample following the first DSC upscan is independent of the initially applied pressure during compression.

### Structural response

**Figure 4:**
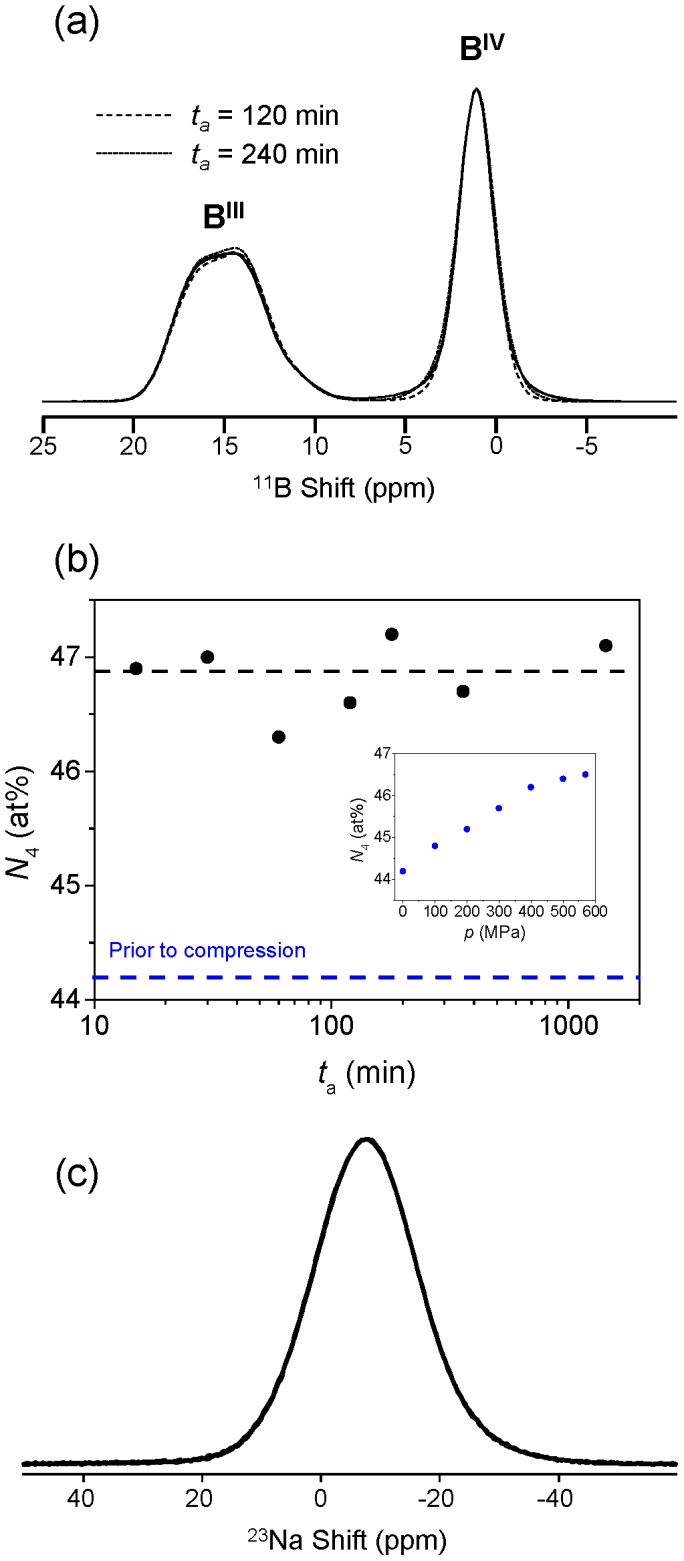
Structural response to annealing. (a) Solid state ^11^B MAS NMR spectra at 16.4 T of the samples compressed at 570 MPa (solid line) and then annealed for various durations (*t*_a_) at 0.9*T*_g_ = 688 K. (b) Evolution of fraction of tetrahedral to total boron (*N*_4_), which is obtained through integration of peaks in Fig. 4a, with annealing duration. Inset: impact of isostatic pressure on *N*_4_ in absence of annealing (corresponding ^11^B spectra are shown in Supplementary Figure 2a). The error of *N*_4_ is ±0.5 at% for the variable pressure series (inset) and ±1 at% for the variable annealing time study. The latter has higher error due to changes in lineshape and thus more difficulty in fitting the ^11^B MAS NMR data. (c) Solid state ^23^Na MAS NMR spectra at 16.4 T of the samples compressed at 570 MPa and then annealed for various durations at 0.9*T*_g_ = 688 K.

^11^B magic-angle spinning (MAS) NMR spectra obtained at 16.4 T (700 MHz) for the 0.57 GPa compressed glass annealed for different durations are shown in Fig. 4a. These spectra are characterised by a broad peak centered at +15 ppm, corresponding to B^III^ sites, and a relatively narrow peak centered around +2 ppm, corresponding to B^IV^ sites. The spectra vary slightly with annealing time, which reflects minor changes in either the relative proportions of B^III^ and B^IV^, or changes in bond angles and distances involving boron and oxygen. We quantify these differences by accurate simulation of the spectral lineshapes and subsequent determination of the fraction of tetrahedral to total boron (*N*_4_) through integration. The simulation parameters are given in Supplementary Table S1, and two examples of the deconvolution are shown in Supplementary Figure S2. Prior to annealing, we find that with increasing isostatic pressure, *N*_4_ increases from 44.2 to 46.5 at% (inset of Fig. 4b and Supplementary Figure S3a). *N*_4_ also increases upon compression in pure B_2_O_3_ glass^17^ and in boron-containing multicomponent E-glass^18^. In Fig. 4b we observe an interesting phenomenon, viz., *N*_4_ of the compressed sample remains almost constant within the error range when extending the annealing time up to 25 hours at the annealing temperature of 688 K (0.9*T*_g_) and at the ambient pressure. In other words, the glass structure, as defined by boron coordination number, does not return to its original state prior to compression upon annealing. Although *N*_4_ does not appear to change, the^11^B MAS NMR spectra do show differences with annealing time. For example, the base of the tetrahedral boron resonances in Fig. 4a exhibit some annealing time dependence, signifying a change in environment of the B^IV^ groups, possibly due to changes in bond angle distribution around these boron atoms. The B^III^ features in Fig. 4a do not show much change with annealing time, indicating that annealing has little impact on B-O bond distance or angles for these structural elements.

Supplementary Figure S3b shows ^23^Na MAS NMR spectra of the samples prior to annealing. We see a small but systematic increase in frequency shift with increasing pressure, which is due to a decrease in the mean Na–O bond distance upon compression^18^. Fig. 4c shows the ^23^Na MAS NMR spectra for the compressed-annealed glasses. These MAS NMR spectra overlap completely, and to further study the sodium speciation, ^23^Na triple-quantum magic-angle spinning (3QMAS) NMR data were also collected. Isotropic projections from these data (Supplementary Figure S4a) show very little change with annealing time, and thus, the estimated quadrupolar coupling product (P_Q_) and isotropic chemical shifts (δ_iso_) for ^23^Na also do not indicate any detectable change in sodium environment in the compressed glass upon annealing (Supplementary Figure S4b). This suggests that the Na–O bond length does not change with annealing time, and hence it does not determine the recovery of the macroscopic properties upon annealing.

## Discussion

Our work has shown that upon annealing at 0.9*T*_g_ of a soda lime borate glass compressed up to ~0.6 GPa, the pressure-induced change in CN remains unchanged while the pressurised values of macroscopic properties such as density, refractive index, and hardness are relaxing. This suggests that the pressure-induced changes in macroscopic properties of such glasses are not attributed to changes in the short-range order, in agreement with previous studies at relatively low pressure^30^. In sodium boroaluminosilicate glasses it has been shown that while pressure-induced density changes are related to changes in boron coordination, the relatively small difference in partial molar volume of the BO_4_ and BO_3_ structural units cannot account for the dominant part of the density change^18^. Instead it was suggested that the shortening of the Na-O bond upon compression is a more likely factor governing the density change^18^,^31^. Such changes were observed at considerably higher pressures than the current study and thus may not in fact account for the findings reported herein. For example, Allwardt *et al.* reported a change in ^23^Na chemical shift of 3.5 ppm at 10 GPa, roughly corresponding to a 0.005 nm decrease in Na-O distance^32^. The chemical shifts for sodium in these soda-lime borates vary slightly with annealing, but within roughly 1–2 ppm and due to measurement uncertainty, appear to indicate a negligible contribution of Na-O bond distance changes to the density change. Shannon & Prewitt^33^ indicate that Na-O bonds should be more compressible than Ca-O bonds. Since Na-O bonds do not change in the detectable range, the contribution of Ca-O bond contraction under pressure could thus likely be ruled out, and therefore the expansion of these bonds does not occur upon annealing, i.e., should not be the reason for the density decrease.

Although boron speciation and modifier-oxygen bond lengths do not change with increased annealing time, density partially decreases. This might be explained as follows. In addition to coordination numbers and bond distances, bond angle distributions (B-O-B and O-B-O) and superstructures (e.g., boroxol rings^34^) may change upon annealing^16^. The change in bond angle and configuration of B^IV^ groups with annealing time could be related to the evolution of the B^IV^ resonance around the frequency shift of +2 ppm in the ^11^B NMR spectra (Fig. 4a). By isostatic compression, not only the connections of neighbored BO_4_ tetrahedra, but possibly also the linkages between boroxol rings, become stronger or tighter, and this is accompanied by the narrowing down of the bond angle distribution. Consequently, the total potential energy in the glass decreases with increasing the compression, which is why the glass transition overshoot becomes larger upon DSC upscanning (see Fig. 2a). Once the compressed glass is subjected to annealing around *T*_g_, the “tightened state” of glass will relax, leading to the decrease of density and hardness. However, the thermal energy at 0.9*T*_g_ could be insufficient for breaking the B-O bonds in favor of formation of BO_3_ units, despite the fact that structural relaxation generally occurs even at temperatures below 0.9*T*_g_^35^. It should be noted that changes in boron speciation due to annealing at 0.92*T*_g_ have previously been reported for non-compressed borosilicate glasses^36^ and the thermomechanically trapped state of the glass studied herein thus appears to be different from that of the thermally trapped glass.

It is known that the *α* relaxation is decoupled from the *β* and *γ* relaxation below *T*_g_^37^. Generally, for network glass systems, the *α* relaxation is dominated by the change of the network connectivity (e.g., the boron speciation), and the *β* relaxation is controlled by the local motion of structural units (e.g., the change of bond angles). In this context, it is understandable that the density relaxes upon annealing slightly below *T*_g_, whereas the CN remains unchanged since the former is of the *β* relaxation feature requiring low temperature and the latter is of the *α* relaxation feature demanding the temperature to exceed *T*_g_. According to literature the B^III^-to-B^IV^ ratio increases with increasing temperature above *T*_g_^36^. Thus, it is expected that the thermomechanically induced B^IV^ units would be converted to B^III^ units when the temperature is well above *T*_g_. In contrast to the density decay, the hardness is fully recovered by annealing at 0.9*T*_g_ to the original value more quickly, as shown in Fig. 1. This could be related to the dominant contribution of the *γ* relaxation process, or to contributions of hydrated structure relaxations of a near surface volume (OH-groups and molecular water) to the relaxation of hardness. The latter has been found recently to proceed more rapidly than both *α* and *β* relaxations^38^. This implies that the hardness relaxation be governed by the fast local network relaxation of the surface layer^39^.

The irreversibility of the thermomechanically induced change in boron speciation upon annealing at 0.9*T*_g_ implies that the free energy barrier for forming a given structural state (i.e., boron speciation) can be overcome not only chemically, but also thermomechanically. This allows for tailoring of both microscopic and macroscopic properties, since the increased network connectivity that remains after annealing should increase, e.g., chemical durability and thermal shock resistance. To further understand this effect, we consider the enthalpy landscape view of glasses^40^. At high temperatures (i.e., well above *T*_g_), the system can flow freely among its configurational microstates, corresponding to the case of an ergodic, equilibrium liquid. As the system is cooled, the configurational transitions occur less frequently owing to the loss of thermal energy. At the glass transition, there is a continuous breakdown of ergodicity as the system gradually becomes trapped in a subset of the available configurational phase space known as a “metabasin”, i.e., a group of configurations that are mutually accessible at a given temperature and for a given observation time^41^. The relatively slow configurational transitions among different metabasins typically involve a concerted series of such transitions. During annealing of the borate glass in this work, there is more thermal energy available for the system to sample the phase space. However, since there is no decrease of *N*_4_ during annealing, the boron coordination state is thermomechanically trapped in a basin in the energy landscape as a consequence of the isostatic compression although the macroscopic properties relax on the time scale of the annealing time. This kinetic effect is caused by a high activation barrier in the enthalpy landscape, preventing the boron coordination number from decreasing. Another possibility is the lack of a thermodynamic driving force for the coordination change. According to the random pair model of Gupta, the equilibrium *N*_4_ value of the studied composition is 49.2 at%^42^,^43^. Hence, the *N*_4_ of the compressed glass is closer to the ambient equilibrium value than that of the as-prepared glass (see inset of Fig. 4b), i.e., there is no thermodynamic driving force to convert B^IV^ into B^III^ units.

## Methods

### Sample preparation

Glass with composition (in mol%) of 25Na_2_O – 10CaO – 65B_2_O_3_ was prepared using melt-quenching technique in an inductively heated furnace, as described in details elsewhere^25^,^43^. Sample rods (4 × 4 × 30 mm^3^) were then isostatically compressed using a cold seal pressure vessel under argon gas. For details of the setup, see Ref. 25. The samples were heated under pressure to a temperature around *T*_g_ + 20 K (*T*_g_ = 764 K), followed by equilibration at this temperature for ~3 min, and finally cooled to room temperature at an initial cooling rate of ~3 K/min. The experiments were performed at the following pressures (*p*): 0.1, 100, 200, 300, 400, 500, and 570 MPa. Relaxation studies were performed on the sample compressed at 570 MPa. This was done by isothermal heat-treatment at 0.9*T*_g_ = 688 K for the following durations (*t*_a_): 15, 30, 60, 120, 180, 360, and 1440 min.

### Physical property measurements

Density (*ρ*) was measured in ethanol using Archimedes' principle. Vickers hardness (*H*_V_) was determined using a micro-indentor (HMV2000, Shimadzu) operated at a load of 9.81 N at ambient conditions using a dwell time of 15 s. The diagonals of the indentation were measured using a 3D laser scanning microscope (VK-9700K, Keyence). Refractive index (*n*) of samples before and after the DSC measurements (see below) were performed at 633 nm using a low range Precision Refractometer (Bausch & Lomb).

### Differential scanning calorimetry

Isobaric heat capacities (*C*_p_) of the investigated samples were determined using a differential scanning calorimeter (DSC Netzsch 404C). DSC runs included measurements of the baseline (two empty Pt–Rh crucibles, 6 mm in diameter, 0.1 mm wall thickness covered with a lid), a sapphire standard (with one crucible containing the standard and the other empty), and finally the sample (with one crucible containing the sample and the other empty). The glass samples were polished to within 1 μm to ensure an accurate fit with the bottom of the crucible and to reach a mass comparable to that of the sapphire standard (~56 mg). Calorimetry was performed under argon flow of 40 ml/min and the glasses were placed on the DSC sample holder at room temperature. Subsequently they were heated to an initial temperature of 313 K, held isothermally for 15 min, and then heated further at a rate of 10 K/min to a temperature 60 K above the *T*_g_ in order to fully relax the sample. At the next step, the sample was cooled at a rate of 10 K/min to 313 K, and then held for one hour prior to the following heating, which occurred at a rate of 10 K/min.

### Solid state NMR

^11^B and ^23^Na MAS NMR experiments were conducted at 16.T using a commercial spectrometer and MAS NMR probes. Resonance frequencies for ^11^B and ^23^Na at this external magnetic field strength were 224.51 and 185.10 MHz, respectively. Samples were crushed using an agate mortar and pestle, packed into 3.2 mm zirconia rotors and spun at frequencies of nominally 20 kHZ. ^11^B and ^23^Na MAS NMR spectra were collected using short radio-frequency pulses (0.6 μs, equivalent to π/12 tip angles), relaxation delays of 2 seconds and signal averaging of 1000 to 2000 acquisitions. Data were frequency referenced to aqueous boric acid at 19.6 ppm and aqueous NaCl at 0 ppm for ^11^B and ^23^Na, respectively. ^11^B MAS NMR spectra were fit using DMfit^44^ to reproduce B^III^ and B^IV^ lineshapes, and *N*_4_ values were determined from integration of these resonances and with consideration of a small correction in B^IV^ intensity due to overlapping satellite transitions^45^. ^23^Na 3QMAS experiments were conducted also at 16.4 T (185.10 MHz resonance frequency) using a 3.2 mm MAS NMR probe and sample spinning of 20 kHz. The pulse sequence used two hard rf pulses (2.3 and 0.9 μs), followed by a z-filter echo using a delay of 10 μs and a soft reading pulse of 15 μs.

## Author Contributions

Y.Z.Y. conceived the study. M.M.S., R.E.Y., S.S., M.P. and U.B. prepared the samples, performed the measurements, and contributed to analysis of the data. M.M.S. and Y.Z.Y. wrote the manuscript with inputs from R.E.Y., J.D., H.B. and J.C.M. All authors were involved in the discussions.

## Supplementary Material

Supplementary Information Irreversibility of Pressure Induced Boron Speciation Change in Glass

## References

[b1] Dubrovinsky L., Dubrovinskaia N., Prakapenka V. B. & Abakumov A. M. Implementation of micro-ball nanodiamond anvils for high-pressure studies above 6 Mbar. Nat. Commun. 3, 1163 (2012). 23093199 10.1038/ncomms2160 PMC3493652

[b2] Yang W. G. *et al.* Coherent diffraction imaging of nanoscale strain evolution in a single crystal under high pressure. Nat. Commun. 4, 1680 (2013). 23575684 10.1038/ncomms2661 PMC3644065

[b3] Mao H. K. *et al.* Phonon density of states of iron up to 153 Gigapascals. Science 292, 914–916 (2001). 11340201 10.1126/science.1057670

[b4] Mishima O., Calvert L. D. & Whalley E. An apparently first-order transition between two amorphous phases of ice induced by pressure. Nature 314, 76–78 (1985).

[b5] Greaves G. N. *et al.* Identifying vibrations that destabilize crystals and characterize the glassy state. Science 308, 1299–1302 (2005). 15919990 10.1126/science.1109411

[b6] Elliot S. R. Physics of Amorphous Materials (Wiley, New York, 1988).

[b7] Yarger J. L. *et al.* Al coordination changes in high-pressure aluminosilicate liquids. Science 270, 1964–1967 (1995).

[b8] Guthrie M. *et al.* The formation and structure of a dense octahedral glass. Phys. Rev. Lett. 93, 115502 (2004). 15447351 10.1103/PhysRevLett.93.115502

[b9] Meng Y. *et al.* The formation of sp(3) bonding in compressed BN. Nat. Mater. 3, 111–114 (2004). 14743214 10.1038/nmat1060

[b10] Monaco A. *et al.* Effect of densification on the density of vibrational states of glasses. Phys. Rev. Lett. 97, 135501 (2006). 17026042 10.1103/PhysRevLett.97.135501

[b11] Bouhadja M., Jakse N. & Pasturel A. Structural and dynamic properties of calcium aluminosilicate melts: A molecular dynamics study. J. Chem. Phys. 138, 224510 (2013). 23781808 10.1063/1.4809523

[b12] Angell C. A., Cheeseman P. A. & Tamaddon S. Pressure enhancement of ion mobilities in liquid silicates from computer simulations studies to 800 kbar. Science 218, 885–887 (1982). 17807142 10.1126/science.218.4575.885

[b13] Klug D. D. *et al.* *Ab initio* molecular dynamics study of the pressure-induced phase transformations in cristobalite. Phys. Rev. B 63, 104106 (2001).

[b14] Huang L. P. *et al.* Transformation pathways of silica under high pressure. Nat. Mater. 5, 977–981 (2006). 17086171 10.1038/nmat1760

[b15] Bauchy M. & Micoulaut M. Transport anomalies and adaptative pressure-dependent topological constraints in tetrahedral liquids: evidence for a reversibility window analogue. Phys. Rev. Lett. 110, 095501 (2013). 23496720 10.1103/PhysRevLett.110.095501

[b16] Grimsditch M., Polian A. & Wright A. C. Irreversible structural changes in vitreous B_2_O_3_ under pressure. Phys. Rev. B 54, 152–155 (1996). 10.1103/physrevb.54.152 9984241

[b17] Lee S. K. *et al.* Probing of bonding changes in B_2_O_3_ glasses at high pressure with inelastic X-ray scattering. Nat. Mater. 4, 851 (2005).

[b18] Wu J. *et al.* Structural response of a highly viscous aluminoborosilicate melt to isotropic and anisotropic compressions. J. Chem. Phys. 131, 104504 (2009).

[b19] Wondraczek L., Sen S., Behrens H. & Youngman R. E. Structure-energy map of alkali borosilicate glasses: Effects of pressure and temperature. Phys. Rev. B 76, 014202 (2007).

[b20] Carini Jr G., Gilioli E., Tripodo G. & Vasi C. Structural changes and elastic characteristics of permanently densified vitreous B_2_O_3_. Phys. Rev. B 84, 024207 (2011).

[b21] Lee S. K., Mibe K., Fei Y. W., Cody G. D. & Mysen B. O. Structure of B_2_O_3_ Glass at High Pressure: A ^11^B Solid-State NMR Study. Phys. Rev. Lett. 94, 165507 (2005). 15904245 10.1103/PhysRevLett.94.165507

[b22] Brazhkin V. V. *et al.* Structural Transformations and Anomalous Viscosity in the B_2_O_3_ Melt under High Pressure. Phys. Rev. Lett. 105, 115701 (2010). 20867586 10.1103/PhysRevLett.105.115701

[b23] Nicholas J. D., Youngman R. E., Sinogeikin S. V., Bass J. D. & Kieffer J. Structural changes in vitreous boron oxide. Phys. Chem. Glasses 44, 249–251 (2003).

[b24] Lee S. K., Eng P. J., Mao H.-K., Meng Y. & Shu J. F. Structure of Alkali Borate Glasses at High Pressure: B and Li *K*-Edge Inelastic X-Ray Scattering Study. Phys. Rev. Lett. 98, 105502 (2007). 17358545 10.1103/PhysRevLett.98.105502

[b25] Striepe S. *et al.* Elastic and micromechanical properties of isostatically compressed soda-lime-borate glasses. J. Non-Cryst. Solids 364, 44–52 (2013).

[b26] Hodge I. M. Enthalpy relaxation and recovery in amorphous materials. J. Non-Cryst. Solids 169, 211–266 (1994).

[b27] Yue Y. Z., Wondraczek L., Behrens H. & Deubener J. Glass transition in an isostatically compressed calcium metaphosphate glass. J. Chem. Phys. 126, 144902 (2007). 17444738 10.1063/1.2719194

[b28] Yue Y. Z., von der Ohe R. & Jensen S. L. Fictive temperature, cooling rate, and viscosity of glasses. J. Chem. Phys. 120, 8053–8059 (2004). 15267724 10.1063/1.1689951

[b29] Yue Y. Z. & Angell C. A. Clarifying the glass-transition behaviour of water by comparison with hyperquenched inorganic glasses. Nature 427, 717–720 (2004). 14973480 10.1038/nature02295

[b30] Wolf G. H. & McMillan P. F. Pressure effects on silicate melt – Structure and properties. Rev. Mineral. 32, 505–561 (1995).

[b31] Wondraczek L., Krolikowski S. & Behrens H. Kinetics of pressure relaxation in a compressed alkali borosilicate glass. J. Non-Crys. Solids 356, 1859–1862 (2010).

[b32] Allwardt J. R. *et al.* Aluminum coordination and the densification of high-pressure aluminosilicate glasses. Am. Mineral. 90, 1218–1222 (2005).

[b33] Shannon R. D. & Prewitt C. T. Effective ionic radii in oxides and fluorides. Acta Crystallogr. B 25, 925–946 (1969).

[b34] Youngman R. E. & Zwanziger J. W. Network Modification in Potassium Borate Glasses: Structural Studies with NMR and Raman Spectroscopies. J. Phys. Chem. 100, 16720–16728 (1996).

[b35] Ya M., Deubener J. & Yue Y. Z. Enthalpy and anisotropy relaxation of glass fibers. J. Am. Ceram. Soc. 91, 745–752 (2008).

[b36] Sen S., Topping T., Yu P. & Youngman R. E. Atomic-scale understanding of structural relaxation in simple and complex borosilicate glasses. Phys. Rev. B 75, 094293 (2007).

[b37] Deubener J., Yue Y. Z., Bornhöft H. & Ya M. Decoupling between birefringence decay, enthalpy relaxation and viscous flow in calcium boroalumosilicate glasses. Chem. Geol. 256, 298–304 (2008).

[b38] Reinsch S., Müller R., Deubener J. & Behrens H. Internal friction of hydrated soda-lime-silicate glasses. J. Chem. Phys. 139, 174506 (2013). 24206315 10.1063/1.4828740

[b39] Smedskjaer M. M., Mauro J. C. & Yue Y. Z. Prediction of Glass Hardness Using Temperature-Dependent Constraint Theory. Phys. Rev. Lett. 105, 115503 (2010). 20867584 10.1103/PhysRevLett.105.115503

[b40] Stillinger F. H. & Weber T. A. Hidden structure in liquids. Phys. Rev. A 25, 978–989 (1982).

[b41] Gupta P. K. & Mauro J. C. The laboratory glass transition. J. Chem. Phys. 126, 224504 (2007). 17581060 10.1063/1.2738471

[b42] Gupta P. K. In Proceedings of the International Congress on Glass (New Delhi, India, 1986).

[b43] Smedskjaer M. M., Mauro J. C., Sen S. & Yue Y. Z. Quantitative design of glassy materials using temperature-dependent constraint theory. Chem. Mater. 22, 5358–5365 (2010).

[b44] Massiot D. *et al.* Modelling one- and two-dimensional solid-state NMR spectra. Magn. Reson. Chem. 40, 70–76 (2002).

[b45] Massiot D., Bessada C., Coutures J. P. & Taulelle F. A quantitative study of ^27^Al MAS NMR in crystalline YAG. J. Magn. Reson. 90, 231–242 (1990).

