# Supplementary material for: Irreversibility of Pressure Induced Boron Speciation Change in Glass
Source: Sci Rep. 2014 Jan 20;4:3770. doi: 10.1038/srep03770 (PMC3895877; doi:10.1038/srep03770)
Supplement: Supplementary Information — Irreversibility of Pressure Induced Boron Speciation Change in Glass [file srep03770-s1.doc]

**Irreversibility of Pressure Induced Boron Speciation Change in Glass**

Morten M. Smedskjaer,^1^ Randall E. Youngman,^2^ Simon Striepe,^3^ Marcel Potuzak,^2^ Ute Bauer,^4^ Joachim Deubener,^3^ Harald Behrens,^4^ John C. Mauro,^2^ and Yuanzheng Yue^1,*^

^1^ *Section of Chemistry, Aalborg University, DK-9000 Aalborg, Denmark*

^2^ *Science and Technology Division, Corning Incorporated, Corning, NY 14831, USA*

^3^ *Institute of Non-Metallic Materials, Clausthal University of Technology, 38678 Clausthal-Zellerfeld, Germany*

^4^ *Institute of Mineralogy, Leibniz University Hannover, 30167 Hannover, Germany*

**Supplementary Information**

**Supplementary Table S1.** DMfit simulation parameters for the ^11^B MAS NMR spectra in Figure 4 (16.4 T) and Figure S3c (11.7 T), including chemical shift (δ_CS_), quadrupolar coupling (C_Q_), quadrupolar asymmetry parameters (η_Q_), and line broadening (lb). Uncertainties in these fitting parameters are on the order of ±0.2 MHz for C_Q_, ±0.5 ppm for δ_CS_ and ±0.1 for η_Q_. Negative values for lb indicate Gaussian broadening. Quadrupolar asymmetry parameters were fixed for the 16.4 T NMR data to avoid unrealistic and large deviations in this parameter. In addition, two B^IV^ resonances were required to fit the 16.4 T data, due to substantial variation in baseline shape with annealing time.

| ***t_a_*** | **BO_3_(I)** | | | | **BO_3_(II)** | | | | **BO_4_(I)** | | **BO_4_(II)** | |
| --- | --- | --- | --- | --- | --- | --- | --- | --- | --- | --- | --- | --- |
| (min) | δ_CS_ (ppm) | C_Q_ (MHz) | η_Q_ | lb (ppm) | δ_CS_ (ppm) | C_Q_ (MHz) | η_Q_ | lb (ppm) | δ_CS_ (ppm) | %  Gaus-sian | δ_CS_ (ppm) | % Gaus-sian |
| 0 | 19.1 | 2.64 | 0.24 | -2.24 | 16.3 | 2.65 | 0.22 | -2.25 | 1 | 65 | --- | --- |
| 15 | 18.7 | 2.61 | 0.25 | -2.44 | 16.1 | 2.50 | 0.18 | -2.18 | 1.1 | 94 | 1.1 | 74 |
| 30 | 18.8 | 2.64 | 0.25 | -2.27 | 16.6 | 2.51 | 0.18 | -2.19 | 1.1 | 97 | 1.1 | 75 |
| 120 | 18.8 | 2.67 | 0.25 | -2.29 | 16.5 | 2.47 | 0.18 | -2.44 | 1.1 | 95 | 1.5 | 100 |
| 180 | 18.6 | 2.59 | 0.25 | -2.5 | 15.6 | 2.43 | 0.18 | -2.21 | 1.1 | 94 | 1.1 | 74 |
| 240 | 18.9 | 2.66 | 0.25 | -2.18 | 16.7 | 2.50 | 0.18 | -2.12 | 1.1 | 92 | 1.3 | 100 |
| 360 | 18.8 | 2.64 | 0.25 | -2.27 | 16.6 | 2.51 | 0.18 | -2.19 | 1.1 | 97 | 1.1 | 75 |
| 1440 | 18.7 | 2.60 | 0.25 | -2.47 | 15.9 | 2.45 | 0.18 | -2.16 | 1.1 | 92 | 1.1 | 80 |

**Supplementary Figure S1. Pressure history dependence of calorimetric glass transition.** Peak value of the heat capacity during glass transition (*C*_p,peak_) evaluated from the first and second DSC upscans, respectively, is plotted as a function of the applied isostatic pressure (*p*). The compressed glass relaxes in the glass transition region during the first DSC upscan with respect to heat capacity, since the value of *C*_p,peak_ for the second upscan is independent of *p*.

**Supplementary Figure S2.** Deconvolution of solid state ^11^B MAS NMR spectra at 16.4 T of the two samples compressed at 570 MPa and then annealed for *t*_a_ = 15 min or *t*_a_ = 1440 min at 0.9*T*_g_ = 688 K.

**Supplementary Figure S3. Structural response to compression.** Solid state ^11^B and ^23^Na MAS NMR spectra, measured at 11.7 T, of the samples prior to annealing, which have been subjected to isostatic compression at different pressures (*p*). The samples were heated under pressure to a temperature around *T*_g_ + 20 K (*T*_g_ = 764 K), followed by equilibration at this temperature for ~3 min, and finally cooled to room temperature at an initial cooling rate of ~3 K/min. (a) ^11^B MAS NMR spectra showing an increase in the relative fraction of B^IV^ to that of B^III^ groups with increasing pressure. (b) ^23^Na MAS NMR spectra showing a small but systematic increase in chemical shift with increasing pressure. This is due a decrease in the mean Na–O bond distance upon compression. (c) DMfit results for the ^11^B MAS NMR of (i) the soda lime borate glass at 1 atm and (ii) after compression to 570 MPa, showing two distinct B^III^ and one B^IV^ resonance.

(a)

*(Figure S3 continued)*

(b)

(c)

**Supplementary Figure S4. ^23^Na 3QMAS NMR results of compressed-annealed samples.** Solid state ^23^Na 3QMAS NMR spectra were obtained at 16.4 T of the samples compressed at 570 MPa and then annealed for various durations (*t*_a_) at 0.9*T*_g_ = 688 K. (a) Isotropic projections of these data, showing little change in lineshape with annealing time. The number shown next to each curve represents the value of *t*_a_ (in minutes). (b) Dependence of quadrupolar coupling constant (P_Q_) and and isotropic chemical shifts (δ_iso_) for ^23^Na on the annealing time. The dashed lines indicate the values of P_Q_ and δ_iso_ prior to compression. Based on measurements errors of isotropic shifts, the errors of P_Q_ and δ_iso_ are estimated to be ±0.2 MHz and ±1.5 ppm, respectively.

(a)

(b)
